# Supplementary material for: An anti-CRISPR that represses its own transcription while blocking Cas9-target DNA binding
Source: Nat Commun. 2024 Feb 28;15:1806. doi: 10.1038/s41467-024-45987-5 (PMC10901769; doi:10.1038/s41467-024-45987-5)
Supplement: Supplementary file 1 — Supplementary Information [file 41467_2024_45987_MOESM1_ESM.pdf]

Supplementary Information For

An anti-CRISPR that represses its own transcription while blocking Cas9-target DNA binding

Xieshuting Deng[1,2], Wei Sun[1], Xueyan Li[1,2], Jiuyu Wang[1], Zhi Cheng[1,2], Gang Sheng[1,2], Yanli Wang[1,2*]

[1]Key Laboratory of RNA Science and Engineering, Institute of Biophysics, Chinese Academy of Sciences, Beijing 100101, China

[2]College of Life Sciences, University of Chinese Academy of Sciences, Beijing 100049, China

*To whom correspondence may be addressed. E-mail: ylwang@ibp.ac.cn

This file contains:

Supplementary Fig. 1-13
Supplementary Table 1-3

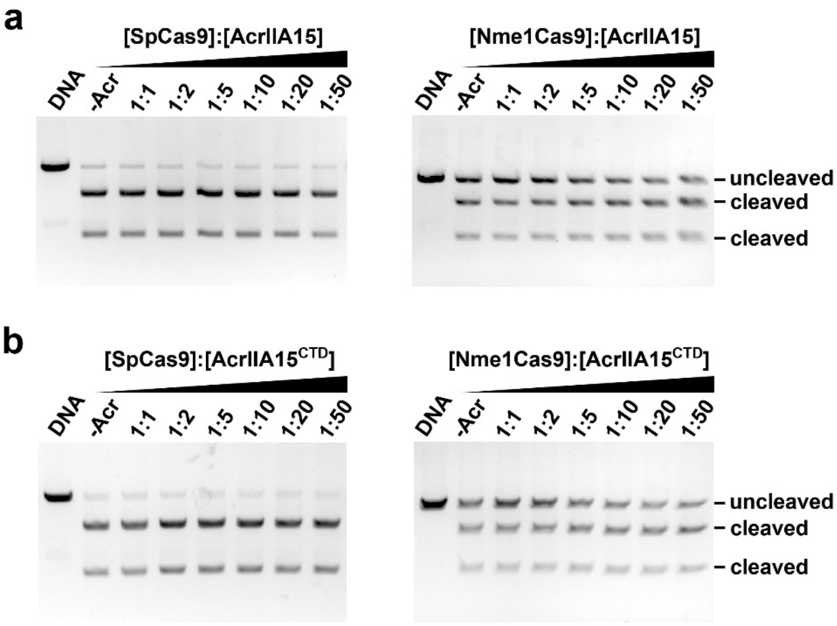

Supplementary Fig. 1 | AcrIIA15 does not inhibit the cleavage activities of SpCas9 and Nme1Cas9.

In vitro cleavage assay of linear plasmid substrates by SpCas9 and Nme1Cas9 in the presence or absence of AcrIIA15 (**a**) or AcrIIA15^{CTD} (**b**). Agarose gels were used for sample separation and stained with ethidium bromide. Source data are provided as a Source Data file.

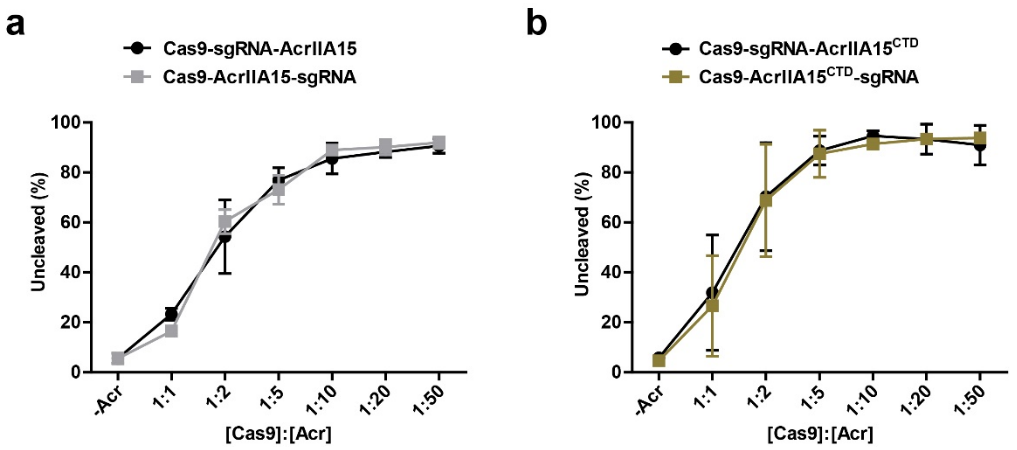

Supplementary Fig. 2 | The order of addition of AcrIIA15 and sgRNA does not impact AcrIIA15's ability to inhibit SaCas9.

In vitro cleavage assay of linear plasmid substrate by SaCas9 with varying molar ratios of full-length AcrIIA15 (**a**) or AcrIIA15^{CTD} (**b**). The Acr was added to SaCas9 before or after the addition of sgRNA. Agarose gels were used for sample separation and stained with ethidium bromide. Afterwards, the gels were subjected to statistical analyses (mean±SD, n=3 independent experiments). Source data are provided as a Source Data file.

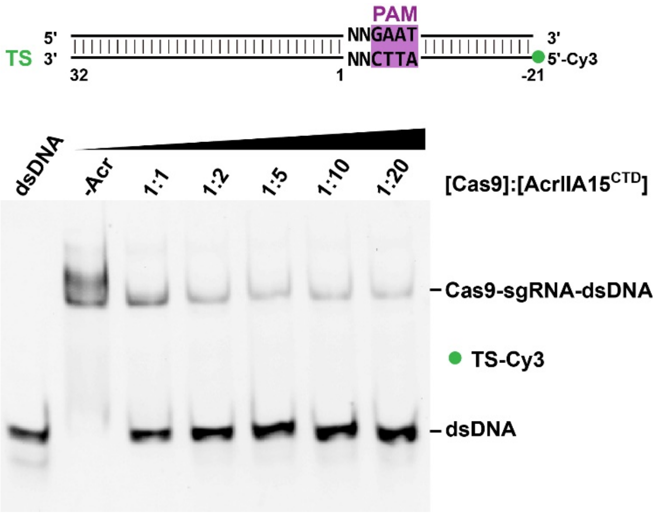

Supplementary Fig. 3 | AcrIIA15^{CTD} blocks target DNA from binding by SaCas9-sgRNA.

(Top) Schematic of the 53-nt dsDNA substrate. The 5'-ends of the target strand (TS) are labeled with Cy3. (Bottom) Different concentrations of AcrIIA15^{CTD} were pre-incubated with the SaCas9-sgRNA binary complex, and then a constant amount of dsDNA was added for further incubation to assess Cas9 DNA binding ability by EMSA. The samples were separated by a 5% native gel and visualized by a FluorChem system. Source data are provided as a Source Data file.

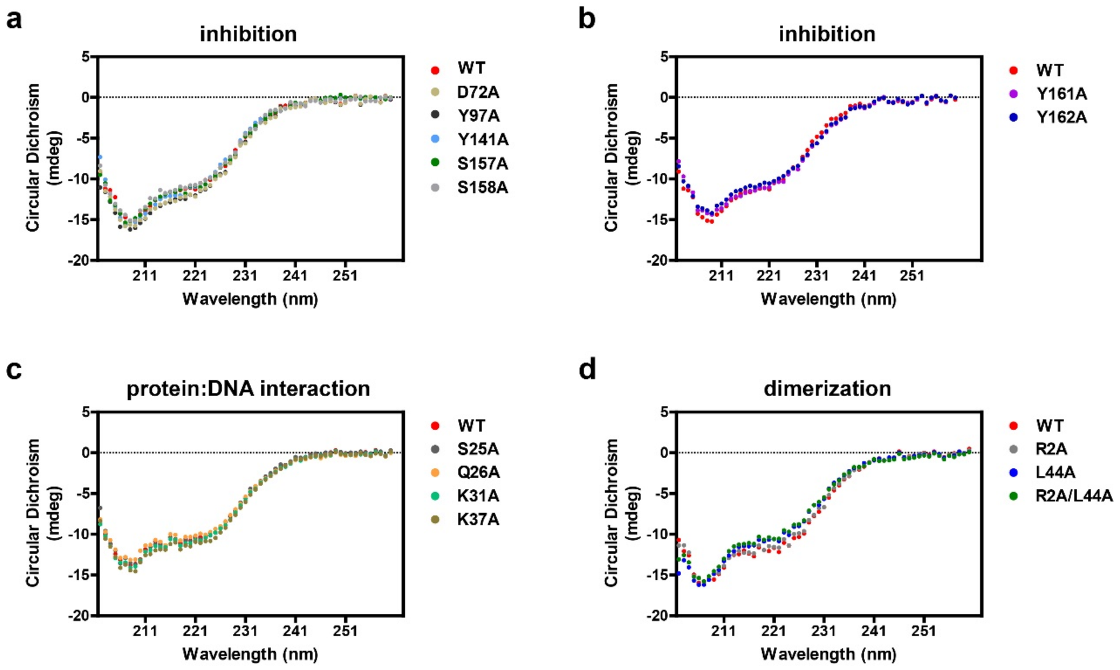

Supplementary Fig. 4 | Circular dichroism spectroscopy scans of wild-type AcrIIA15 and its mutants. a, b, Scans of AcrIIA15-CTD critical for cleavage inhibition. **c, d,** Scans of AcrIIA15 critical for protein:DNA binding (**c**) and dimerization (**d**). Each scan was an average of three replicates.

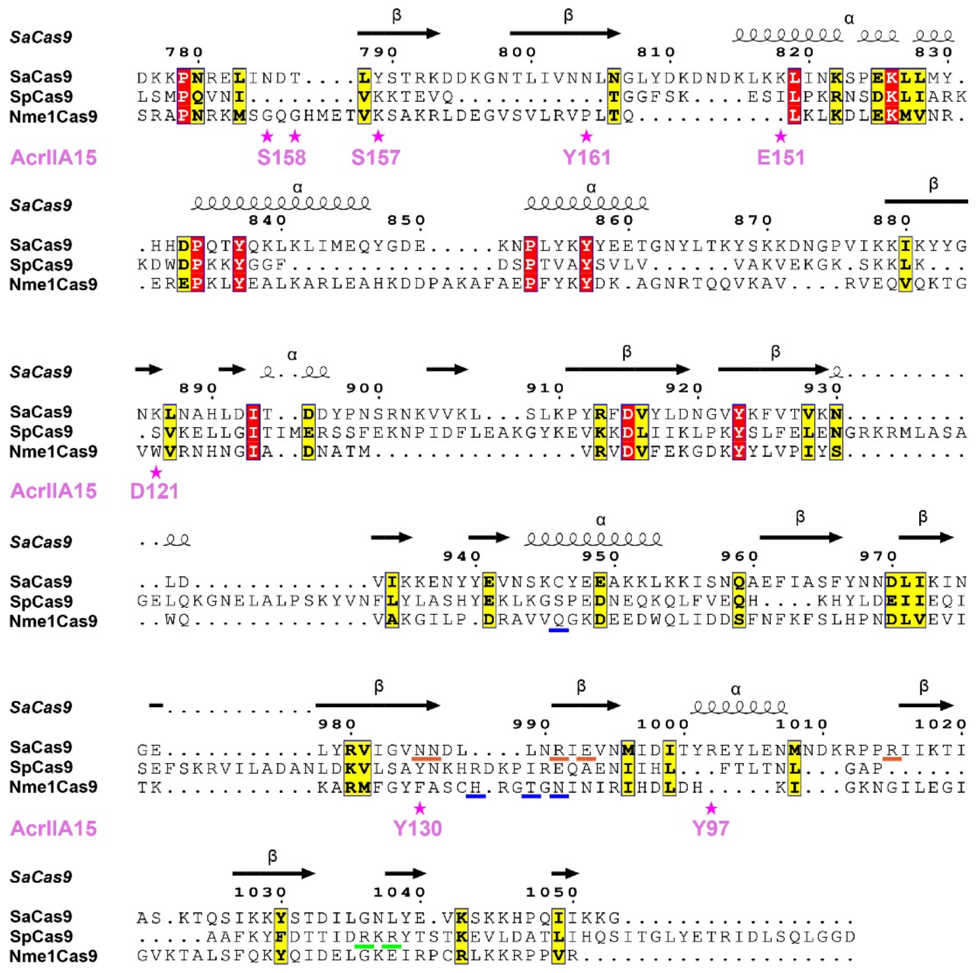

Supplementary Fig. 5 | Sequence alignment of the CTDs of SaCas9, SpCas9 and Nme1Cas9. Residues of SaCas9 critical for AcrIIA15 inhibition are highlighted with magenta star, and underneath are the corresponding AcrIIA15 residues. PAM recognition residues of SaCas9, SpCas9 and Nme1Cas9 are underscored with orange, green, and blue lines, respectively.

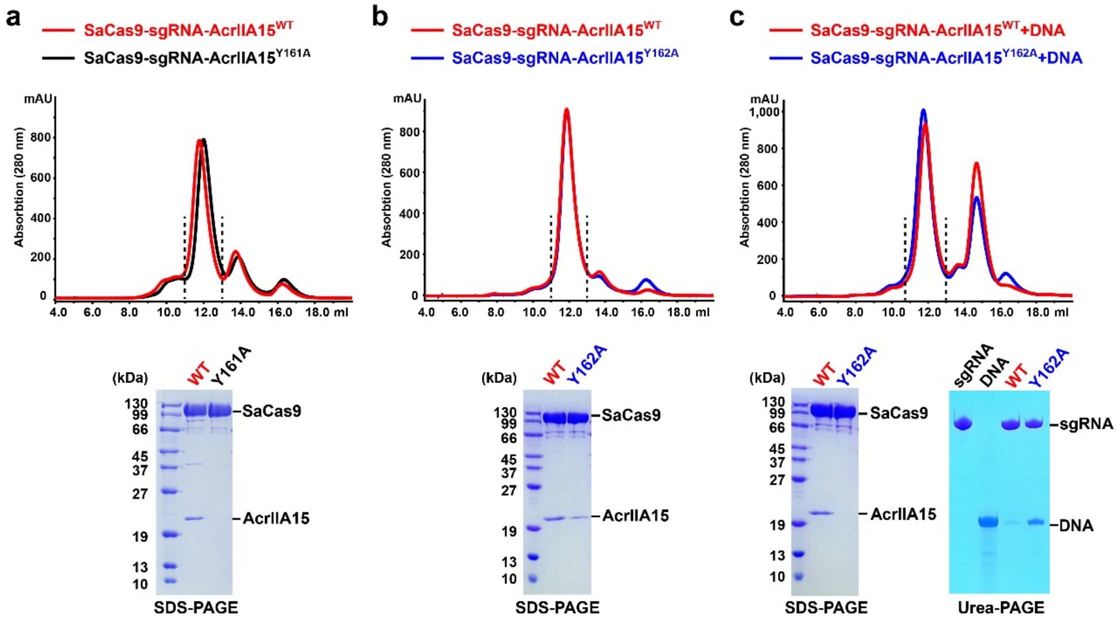

Supplementary Fig. 6 | Residues Y161 and Y162 of AcrIIA15^{CTD} are critical for inhibition of Cas9-dsDNA binding.

a, Overlay of SEC profiles of SaCas9-sgRNA-AcrIIA15^{CTD} complexes for either wild-type AcrIIA15 or Y161A mutant.

b, Overlay of SEC profiles of SaCas9-sgRNA-AcrIIA15^{CTD} complexes for either wild-type AcrIIA15 or Y162A mutant.

c, Overlay of SEC profiles of SaCas9-sgRNA-AcrIIA15^{CTD}+DNA complexes for either wild-type AcrIIA15 or Y162A mutant.

For all the SEC experiments, fractions from the major peaks (highlighted with vertical dashed lines) were characterized by SDS-PAGE or Urea-PAGE gels stained with Coomassie blue and toluidine blue, respectively. Source data are provided as a Source Data file.

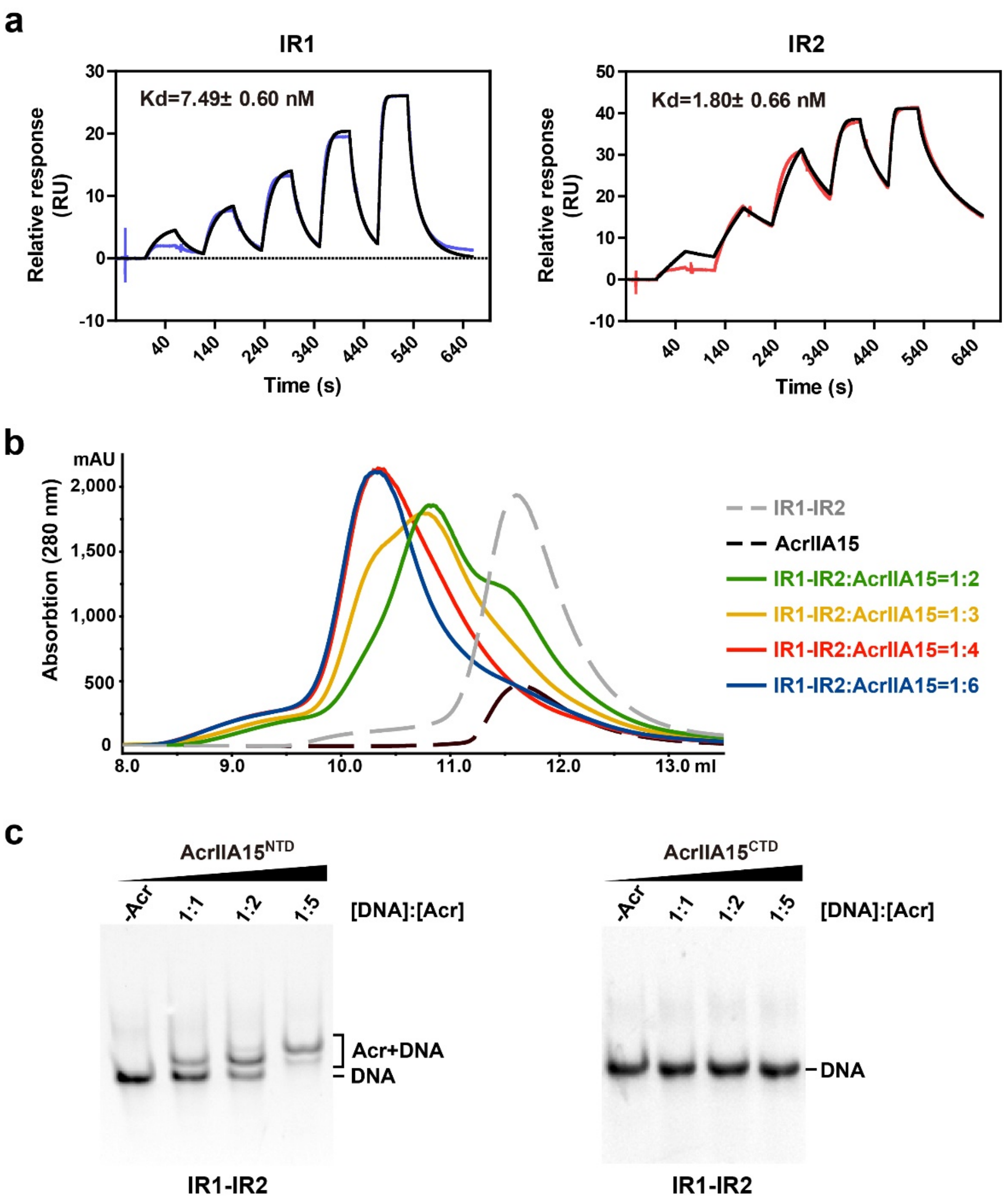

Supplementary Fig. 7 | AcrIIA15^{NTD} governs binding to IR-containing dsDNA.

a, Surface plasmon resonance (SPR) measuring the binding affinities between AcrIIA15 and IR1 or IR2. A representative result was shown, and the dissociation constants (Kd) were calculated from three replicates (mean±SD, n=3). The blue and red curves are experimental traces, and the black curves are fits calculated by Biacore software.

b, SEC measuring the stoichiometric ratio of AcrIIA15 binding to IR1-IR2 promoter DNA.

c, Various molar ratios of dsDNA to AcrIIA15 were incubated together for EMSA. The NTD (left panel) and CTD (right panel) were tested separately. Samples were separated on a 5% native gel stained with ethidium bromide. Source data are provided as a Source Data file.

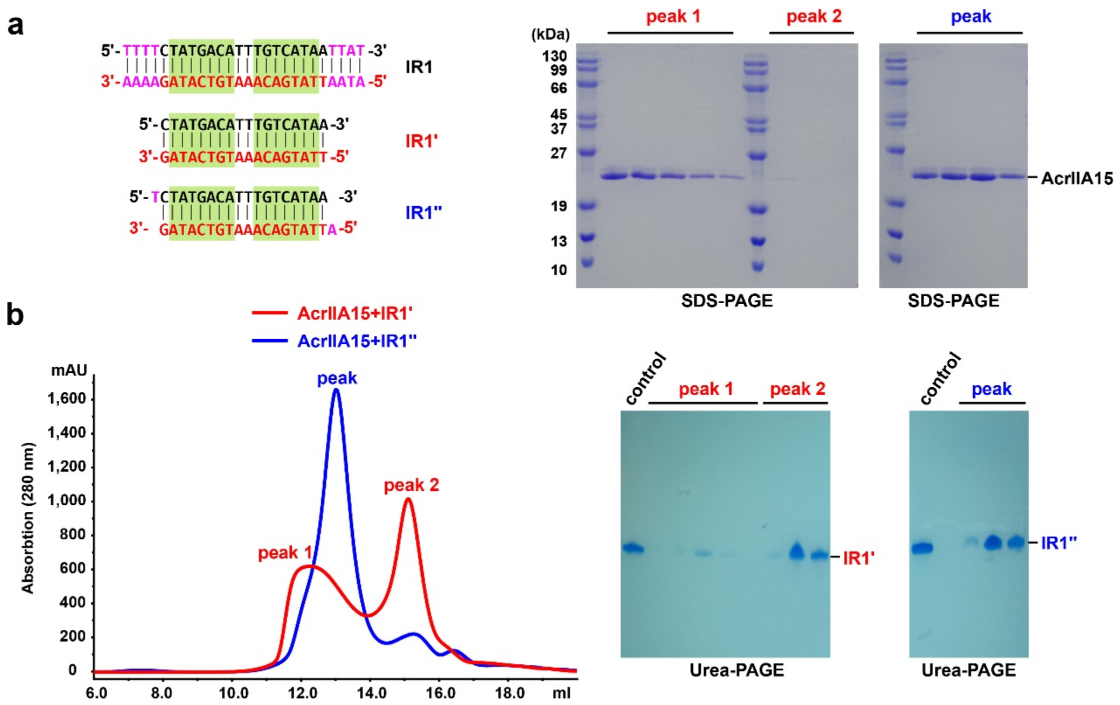

Supplementary Fig. 8 | SEC detecting the minimal length of IR1 used for AcrIIA15-IR1 complex reconstitution.

a, Schematic of IR1 and its two variants, named IR1' and IR1'', used for SEC.

b, Overlay of SEC profiles of AcrIIA15+IR1' and AcrIIA15+IR1''. Fractions from the three labeled peaks were characterized by SDS-PAGE and Urea-PAGE. Source data are provided as a Source Data file.

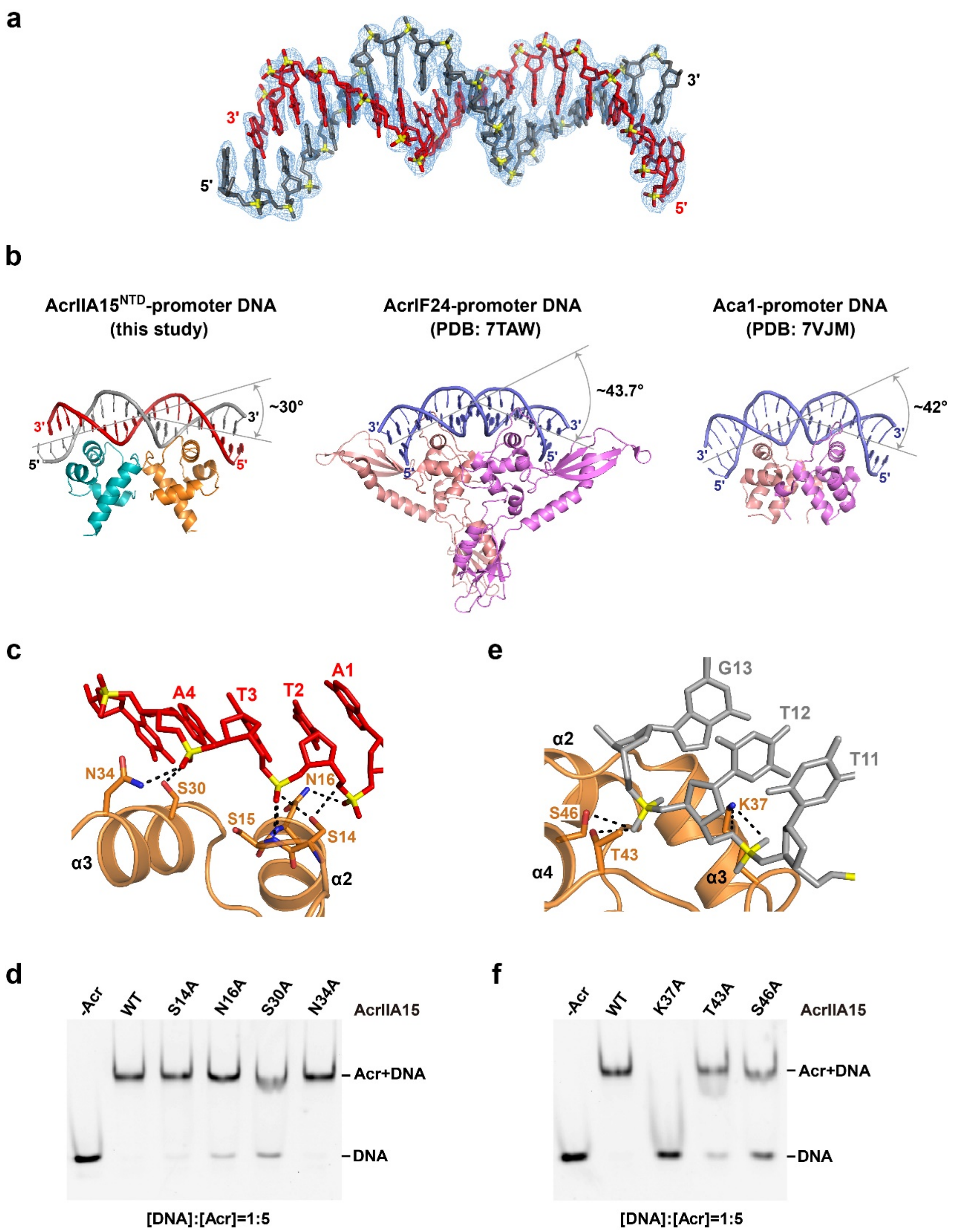

Supplementary Fig. 9 | Characterization of residues of AcrIIA15^{NTD} involved in DNA recognition by EMSA.

a, The 2mFo-Fc electron density map of the DNA in the structure of AcrIIA15^{NTD}-DNA is shown as a blue mesh (contoured at 1.7σ).

b, Different bending conformations of dsDNA caused by AcrIIA15^{NTD}, AcrIF24 and Aca1 protein binding.

c,d, The interactions between amino acids of AcrIIA15^{NTD} and the phosphate backbone of DNA at the region adjacent to 5'-end (**c**) and EMSA characterizing mutations in key

residues of AcrIIA15 involved (**d**).

e,f, The interactions between amino acids of AcrIIA15^{NTD} and the phosphate backbone of DNA at the middle region (**e**) and EMSA characterizing mutations in key residues of AcrIIA15 involved (**f**). Cy3-labeled IR1 dsDNA was used for both EMSA experiments, and EMSA samples were separated by 5% native gel and visualized using a FluorChem system. Source data are provided as a Source Data file.

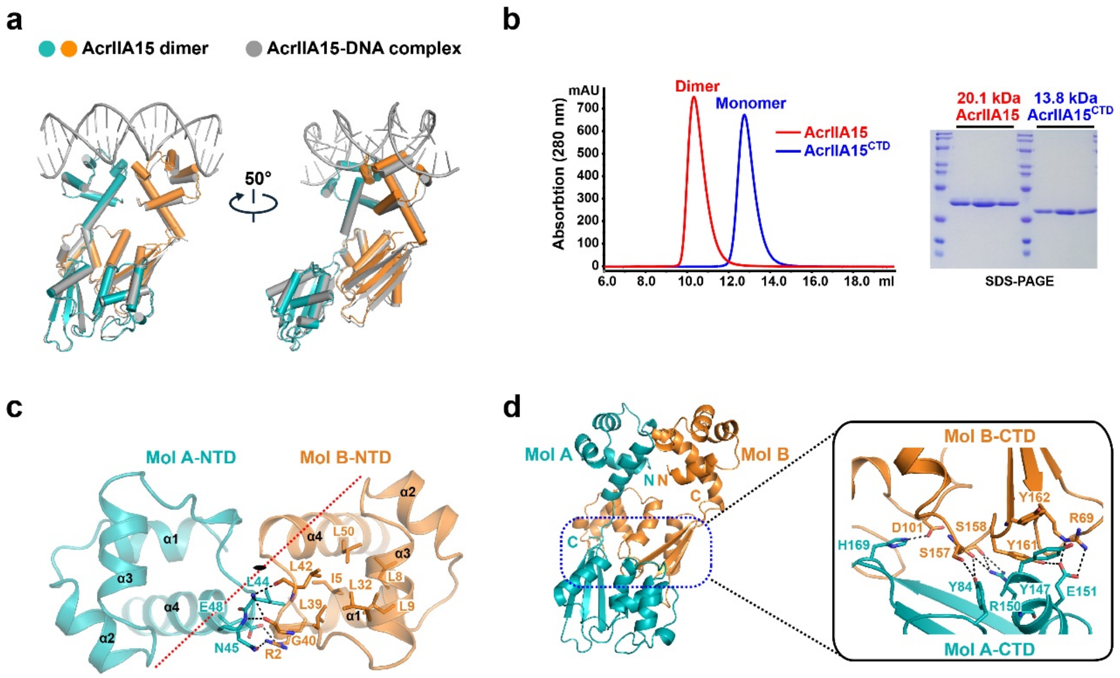

Supplementary Fig. 10 | Comparison of structures of AcrIIA15 with and without DNA bound.

a, Superposition of structures of apo- and DNA-bound AcrIIA15. Apo-AcrIIA15 is colored in teal and orange (monomers A and B, respectively), and the AcrIIA15-DNA complex is colored in gray.

b, SEC profiles detecting the oligomerization states of full-length AcrIIA15 or its isolated CTD. Fractions from the major peaks were evaluated by SDS-PAGE and stained with Coomassie blue.

c, Hydrophobic core of the NTD of AcrIIA15. Only half of the hydrophobic residues are shown for clarity. The dotted red line illustrates two symmetric halves.

d, Interactions between the CTDs of monomer A and monomer B in the structure of apo-AcrIIA15. Source data are provided as a Source Data file.

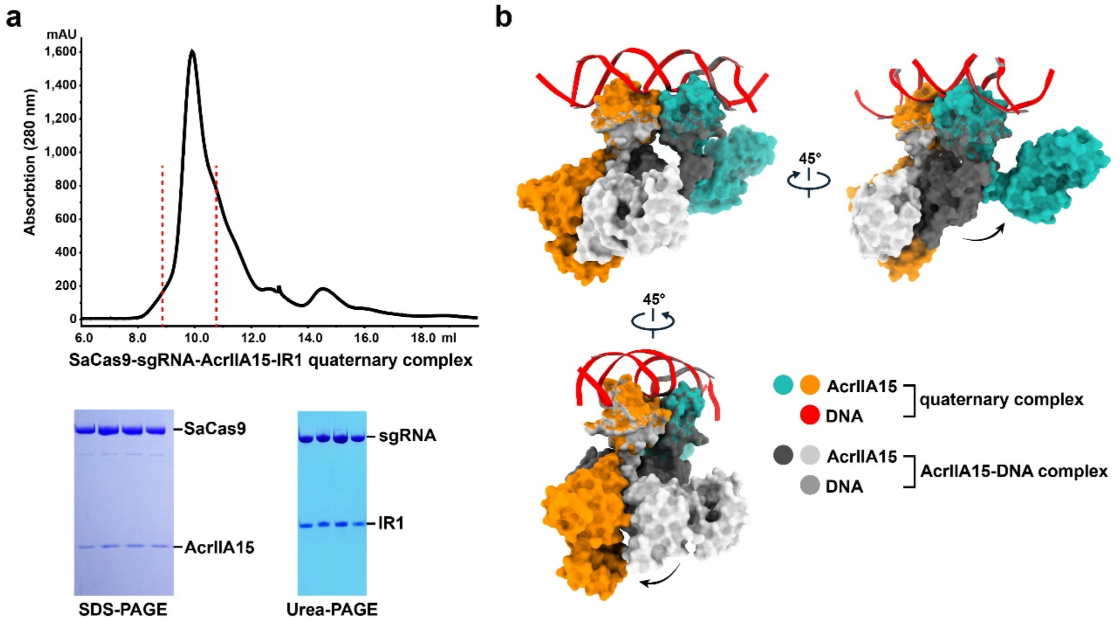

Supplementary Fig. 11 | The SaCas9-sgRNA-AcrIIA15-IR1 complex induces a conformational change in the AcrIIA15 dimer.

a, SEC of the SaCas9-sgRNA-AcrIIA15-IR1 complex. Peak fractions from the area highlighted with vertical red dashed lines were characterized by SDS-PAGE and urea gel.

b, Superposition of structures of the AcrIIA15-DNA binary complex (colored in gray) and the AcrIIA15-DNA moiety in the quaternary complex (colored in teal/orange and red) aligned via the NTD of AcrIIA15. Black arrows indicate the rotational direction of AcrIIA15^{CTD} after binding to SaCas9. Source data are provided as a Source Data file.

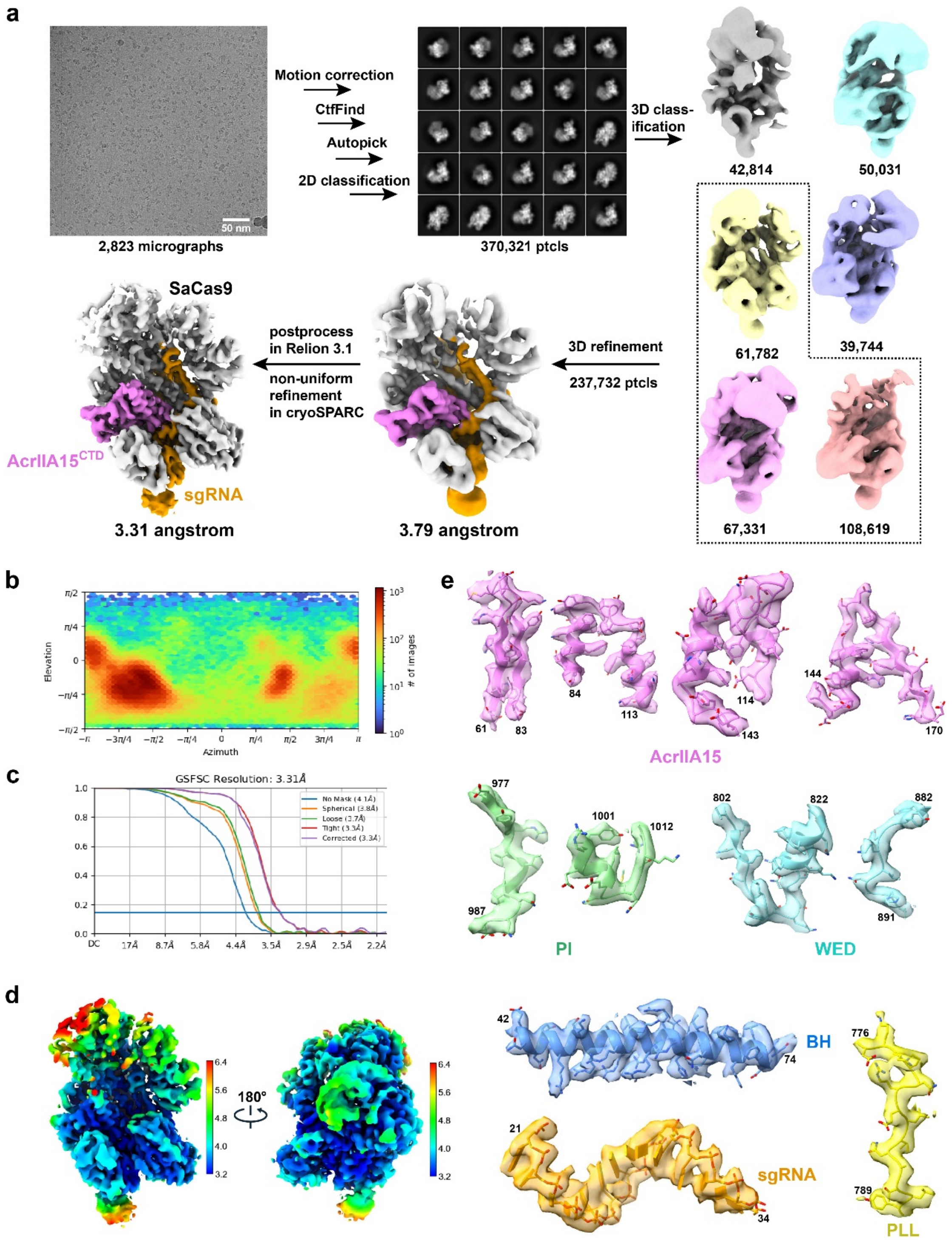

Supplementary Fig. 12 | Single particle Cryo-EM analysis of SaCas9-sgRNA-AcrIIA15^{CTD} ternary complex.

a, Data-processing workflow for the cryo-EM structure of the ternary complex.

b, Angular distribution of particles included in the final 3D reconstruction.

c, The gold standard Fourier shell correlation (FSC) curve of the final density map.

d, Local resolutions of the cryo-EM map as estimated by ResMap.

e, Density maps of AcrIIA15 and related residues of SaCas9 and sgRNA.

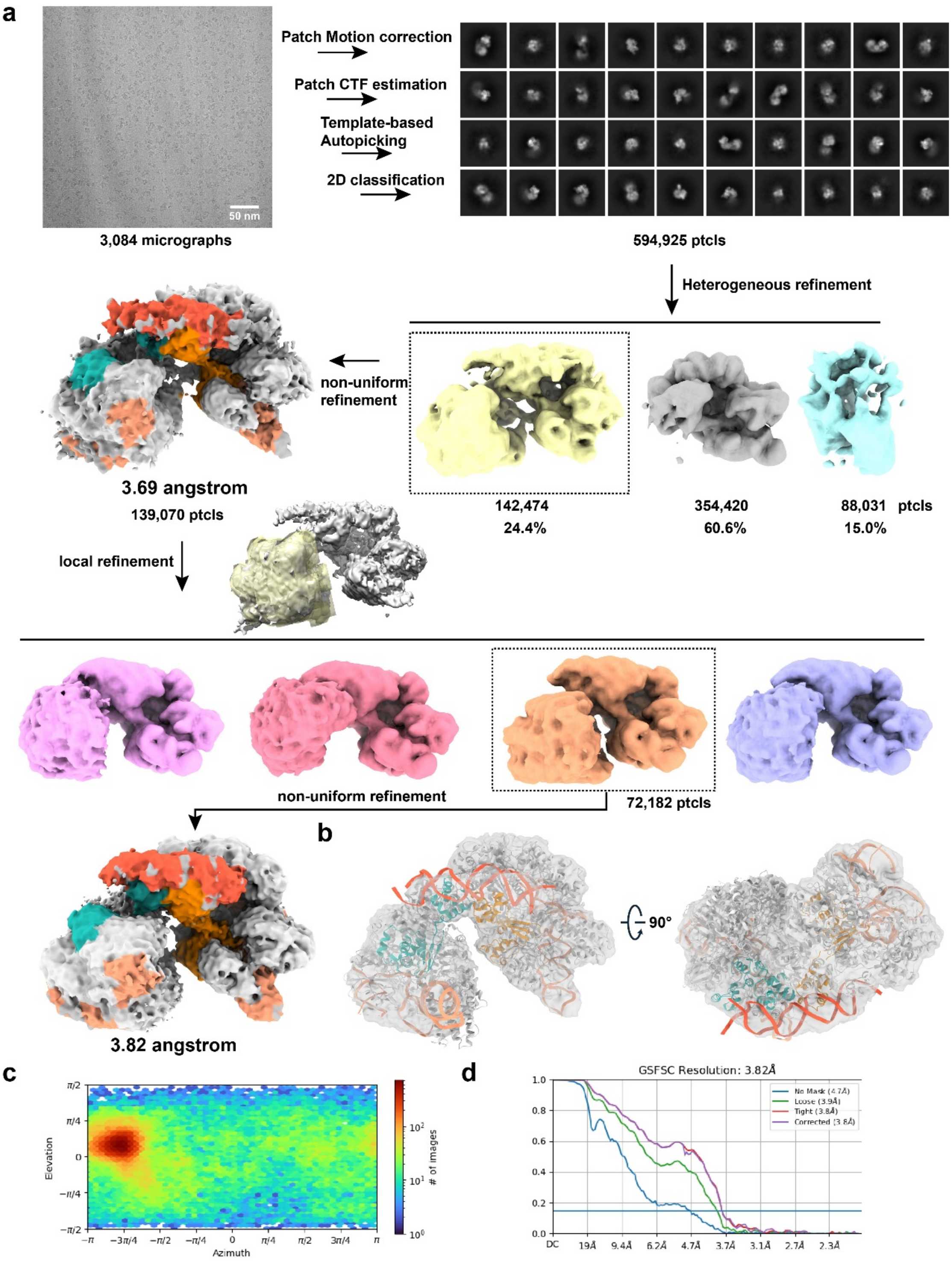

Supplementary Fig. 13 | Single particle Cryo-EM analysis of SaCas9-sgRNA-AcrIIA15-IR1 quaternary complex.

a, Data-processing workflow for the quaternary complex.

b, Density maps and cartoon representation of the quaternary complex.

c, Angular distribution of particles included in the final 3D reconstruction.

d, The gold standard Fourier shell correlation (FSC) curve of the final density map.

Supplementary Table 1. Sequences of nucleic acids used in this paper.

IDENTIFIER	Related figures	Sequence (5’ to 3’)
plasmid target of SaCas9	Figure 1a, 3g; Supplementary Fig. 2	NTS: GGTCTGCTATTTCTATTTAC
		TS: GTAAATAGAAATAGCAGACC
sgRNA for SaCas9	Figure 1-3; Supplementary Fig. 2, 3, 6	GGUCUGCUAUUUCUAUUUACGUUUUAGUACUCUGGAAACAGAAUCUACUAAAACAAGGCAAAAUGCCGUGUUUAUCUCGUCAACUUGUUGGCGAGAUC
Target dsDNA for SEC	Figure 1b; Supplementary Fig. 6	NTS: GGTCTGCTATTTCTATTTACTTGAATAGCAC
		TS: GTGCTATTCAAGTAAATAGAAATAGCAGACC
Inverted repeat (IR1-IR2) for EMSA and SEC	Figure 4a; Supplementary Fig. 7b, 7c	IRs F: ATAATTATGACAAATGTCATAGAAAAGCGTTGACTTATGACGAACGTCATAATATA
		IRs R: TATATTATGACGTTCGTCATAAGTCAACGCTTTTCTATGACATTTGTCATAATTAT
Inverted repeat (IR1) for EMSA, SPR and SEC	Figure 4a, 5e, 6f; Supplementary Fig. 7a, 11	IR1 F: ATAATTATGACAAATGTCATAGAAAA
		IR1 R: TTTTCTATGACATTTGTCATAATTAT
Inverted repeat (IR1) for EMSA (Cy3 labeled DNA)	Figure 5f, 6e; Supplementary Fig. 9d, 9f	IR1 F: Cy3-ATAATTATGACAAATGTCATAGAAAA
		IR1 R: TTTTCTATGACATTTGTCATAATTAT
Inverted repeat (IR2) for EMSA and SPR	Figure 4a; Supplementary 7a	IR2 F: TGACTTATGACGAACGTCATAATATA
		IR2 R: TATATTATGACGTTCGTCATAAGTCA
IR1’’ for crystal growth and SEC	Figure 4b, 4c Supplementary Fig. 8	IR’’ F: ATTATGACAAATGTCATAG
		IR’’ R: TCTATGACATTTGTCATAA
IR1 mutant in A4:T4' and T17: A17' for EMSA	Figure 5e	F: ATAATTGTGACAAATGTCACAGAAAA
		R: TTTTCTGTGACATTTGTCACAATTAT
IR1 mutant in G6:C6' and C15:G15' for EMSA	Figure 5e	F: ATAATTATTACAAATGTAATAGAAAA
		R: TTTTCTATTACATTTGTAATAATTAT
IR1 mutant in four base pairs for EMSA	Figure 5e	F: ATAATTGTTACAAATGTAACAGAAAA
		R: TTTTCTGTTACATTTGTAACAATTAT

A15 promoter for in vivo GFP reporter assays	Figure 5g	ACCAACAAAAATAATTATGACAAATGTCATAGAAAAGCGTTGACTTATGACGAACGTCATAATATAATATAGACATAAGGTAATCACAGGAGGAAATAAA
sgRNA for SpyCas9	Supplementary Fig. 1	GGAAAUUAGGUGCGCUUGGCGUUUUAGAGCUAGAAAUAGCAAGUUAAAAUAAGGCUAGUCCGUUAUCAACUUGAAAAAGUGGCACCGAGUCGGUGCUUC
plasmid target of SpyCas9	Supplementary Fig. 1	NTS: GGAAATTAGGTGCGCTTGGCTGGTATTG
		TS: CAATACCAGCCAAGCGCACCTAATTTCC
sgRNA for Nme1Cas9	Supplementary Fig. 1	GGUCACUCUGCUAUUUAACUUUACGUUGUAGCUCCCUUUCUCGAAAGAGAACCGUUGCUACAAUAAGGCCGUCUGAAAAGAUGUGCCGCAACGCUCUGCCCCUUAAAGCUCCUGCUUUAAGGGGCAUCGUUUAUC
plasmid target of Nme1Cas9	Supplementary Fig. 1	NTS: GGTCACTCTGCTATTTAACTTTACATATGATTTTA
		TS: TAAAATCATATGTAAAGTTAAATAGCAGAGTGACC
Cy3-labeled TS for EMSA	Supplementary Fig. 3	Cy3-CTCAGTGATCCTACTATTCAAGTAAATAGAAATAGCAGACCGATCTGTCATGA
NTS for EMSA	Supplementary Fig. 3	TCATGACAGATCGGTCTGCTATTTCTATTTACTTGAATAGTAGGATCACTGAG
IR1’ for SEC	Supplementary Fig. 8	IR’ F: TTATGACAAATGTCATAG
		IR’ R: CTATGACATTTGTCATAA

Supplementary Table 2. Crystallographic data collection and refinement statistics.

	AcrIIA15	AcrIIA15-DNA	AcrIIA15^{NTD}-DNA
PDB code	8JFO	8JFU	8JFR
Beamline	SSRF BL02U1	SSRF BL19U1	SSRF BL19U1
Space group	$P4_1$	$C222_1$	$P4_3$
**Cell dimensions**			
a, b, c (Å)	88.194; 88.194; 145.649	110.604; 131.388; 234.183	58.712; 58.712; 240.593
α，β，γ (°)	90.00; 90.00; 90.00	90.00; 90.00; 90.00	90.00; 90.00; 90.00
**Data Collection**			
Wavelength (Å)	0.97918	0.97851	0.97851
Resolution (Å)	34.68 - 2.30 (2.34 - 2.30)	50.00 - 3.15 (3.15-3.22)	50.00 - 3.10 (3.10-3.15)
Completeness (%)	99.7(99.4)	99.7(99.7)	99.7(99.9)
Redundancy	5.8（5.3）	5.5 (4.7)	4.6(4.7)
I/σI	21.6 (1.59)	10.0（1.36）	24.9(5.15)
Rmerge (%)	7.2（97.6）	13.1 (84.2)	5.5(28.9)
Rpim (%)	3.1 (44.1)	5.9 (42.4)	2.8(14.8)
**Refinement**			
Resolution (Å)	34.68-2.30	45.13 - 3.15	29.66-3.10
No. Reflections	46108	18412	9394
R_work/R_free (%)	22.23/23.35	25.98/27.37	24.62/25.86
B-factors ($Å^2$)			
Protein	43.85	76.71	58.17
Nucleic acid		66.09	69.85
Ligand			
Water	40.18		
R.m.s. deviation			
Bond lengths (Å)	0.01	0.01	0.01
Bond angles (°)	1.31	1.11	1.29
Number of Atoms			
Protein	5506	5001	1814
Nucleic acid		1546	1546
Water	416		
Ramachandran Plot			
Favored (%)	99.41	98.74	100
Allowed (%)	0.59	1.26	0.00
Outliers (%)	0.00	0.00	0.00

Supplementary Table 3. Cryo-EM data collection, refinement and validation statistics.

	SaCas9-sgRNA-AcrIIA15^{CTD} (PDB: 8JFT; EMDB: EMD-36217)	SaCas9-sgRNA-AcrIIA15-IR1 (PDB: 8JG9; EMDB: EMD-36225)
**Data collection and processing**		
Magnification	130,000	130,000
Voltage (kV)	300	300
Electron exposure (e-/Å^2)	60	60
Defocus range (um)	1.4-1.8	1.0-1.5
Pixel size (Å)	1.04	1.04
Symmetry imposed	C1	C1
Initial particle images (no.)	3,926,680	1,125,640
Final particle images (no.)	237,732	72,182
Map resolution (Å)	3.31	3.82
FSC threshold	0.143	0.143
Map resolution range (Å)	3.2-6.4	
**Refinement**		
Initial model used (PDB code)	5AXW	5AXW
Model resolution (Å)	3.5； 4.0	3.96；8.93
FSC threshold	0.143; 0.5	0.143; 0.5
Map sharpening B factor (Å^2)	-147.1	-65.6
Model composition		
Non-hydrogen atoms	11176	24264
Protein residues	1151	2424
Nucleotides	86	222
Ligands	0	0
Mean B factors (Å^2)		
Protein	149.43	145.19
Nucleotides	141.17	122.65
R.m.s. deviations		
Bond lengths (Å)	0.008	0.009
Bond angles (°)	1.360	1.353
Validation		
MolProbity score	1.73	1.79
Clashscore	11.41	11.46
Poor rotamers (%)	0.90	1.19
Ramachandran Plot		
Favored (%)	97.12	97.10
Allowed (%)	2.88	2.90
Outliers (%)	0.00	0.00
