# Supplementary material for: An anti-CRISPR that represses its own transcription while blocking Cas9-target DNA binding
Source: Nat Commun. 2024 Feb 28;15:1806. doi: 10.1038/s41467-024-45987-5 (PMC10901769; doi:10.1038/s41467-024-45987-5)
Supplement: Supplementary file 3 — Reporting Summary [file 41467_2024_45987_MOESM3_ESM.pdf]

nature portfolio

Corresponding author(s): Yanli Wang

Last updated by author(s): Jan 22, 2024

Reporting Summary

Nature Portfolio wishes to improve the reproducibility of the work that we publish. This form provides structure for consistency and transparency in reporting. For further information on Nature Portfolio policies, see our Editorial Policies and the Editorial Policy Checklist.

Statistics

For all statistical analyses, confirm that the following items are present in the figure legend, table legend, main text, or Methods section.

n/a	Confirmed	
☐	☒	The exact sample size (*n*) for each experimental group/condition, given as a discrete number and unit of measurement
☐	☒	A statement on whether measurements were taken from distinct samples or whether the same sample was measured repeatedly
☒	☐	The statistical test(s) used AND whether they are one- or two-sided<br>*Only common tests should be described solely by name; describe more complex techniques in the Methods section.*
☒	☐	A description of all covariates tested
☒	☐	A description of any assumptions or corrections, such as tests of normality and adjustment for multiple comparisons
☐	☒	A full description of the statistical parameters including central tendency (e.g. means) or other basic estimates (e.g. regression coefficient) AND variation (e.g. standard deviation) or associated estimates of uncertainty (e.g. confidence intervals)
☒	☐	For null hypothesis testing, the test statistic (e.g. *F*, *t*, *r*) with confidence intervals, effect sizes, degrees of freedom and *P* value noted<br>*Give P values as exact values whenever suitable.*
☒	☐	For Bayesian analysis, information on the choice of priors and Markov chain Monte Carlo settings
☒	☐	For hierarchical and complex designs, identification of the appropriate level for tests and full reporting of outcomes
☒	☐	Estimates of effect sizes (e.g. Cohen's *d*, Pearson's *r*), indicating how they were calculated

Our web collection on statistics for biologists contains articles on many of the points above.

Software and code

Policy information about availability of computer code

Data collection	The cryo-EM data were collected using SerialEM (3.8). X-ray diffraction data were collected at beamlines BL02U1 and BL19U1 (Shanghai Synchrotron Radiation Facility).
Data analysis	Cryo-EM datasets were analyzed with MotionCor2(1.4.0), CTFFind(4.1.14), RELION(3.1.3), CryoSPARC(3.3.0). X-ray crystallographic datasets were integrated and scaled using HKL2000 and HKL3000 software. Phasing and refinement were performed in PHENIX(1.9.2) software. The structures were built in COOT(0.9-pre). All structures were visualized in Pymol 2.5.2, UCSF Chimera (1.16), or ChimeraX(1.3). The SPR data were analyzed by Biacore Insight Evaluation (3.0.12.15655). Quantification of gel bands were performed by ImageJ (1.40g), and the graphs were illustrated by GraphPad Prism (v7.04).

For manuscripts utilizing custom algorithms or software that are central to the research but not yet described in published literature, software must be made available to editors and reviewers. We strongly encourage code deposition in a community repository (e.g. GitHub). See the Nature Portfolio guidelines for submitting code & software for further information.

Data

Policy information about availability of data

All manuscripts must include a data availability statement. This statement should provide the following information, where applicable:

- Accession codes, unique identifiers, or web links for publicly available datasets
- A description of any restrictions on data availability
- For clinical datasets or third party data, please ensure that the statement adheres to our policy

The atomic coordinates generated in this study have been deposited in the Protein Data Bank (PDB) under the accession code 8JFO [https://doi.org/10.2210/pdb8JFO/pdb] (apo-AcrIIA15), 8JFU [https://doi.org/10.2210/pdb8JFU/pdb] (AcrIIA15-DNA), 8JFR [https://doi.org/10.2210/pdb8JFR/pdb] (AcrIIA15NTD-DNA), 8JFT [https://doi.org/10.2210/pdb8JFT/pdb] (SaCas9-sgRNA-AcrIIA15CTD) and 8JG9 [https://doi.org/10.2210/pdb8JG9/pdb] (SaCas9-sgRNA-AcrIIA15-IR), the cryo-EM maps generated in this study have been deposited in the Electron Microscopy Data Bank (EMDB) under the accession number EMD-36217 [https://www.ebi.ac.uk/emdb/EMD-36217] (SaCas9-sgRNA-AcrIIA15CTD) and EMD-36225 [https://www.ebi.ac.uk/emdb/EMD-36225] (SaCas9-sgRNA-AcrIIA15-IR). Two previously published atomic coordinates were used in Fig. 2: 5AXW [https://doi.org/10.2210/pdb5AXW/pdb] (SaCas9-sgRNA) and 355D [https://doi.org/10.2210/pdb355D/pdb] (a typical B-form DNA). The gels and fluorescence data generated in this study are provided in the Source Data file. Source data are provided with this paper.

Research involving human participants, their data, or biological material

Policy information about studies with human participants or human data. See also policy information about sex, gender (identity/presentation), and sexual orientation and race, ethnicity and racism.

Reporting on sex and gender	Do not apply.
Reporting on race, ethnicity, or other socially relevant groupings	Do not apply.
Population characteristics	Do not apply.
Recruitment	Do not apply.
Ethics oversight	Do not apply.

Note that full information on the approval of the study protocol must also be provided in the manuscript.

Field-specific reporting

Please select the one below that is the best fit for your research. If you are not sure, read the appropriate sections before making your selection.

☒ Life sciences ☐ Behavioural & social sciences ☐ Ecological, evolutionary & environmental sciences

For a reference copy of the document with all sections, see nature.com/documents/nr-reporting-summary-flat.pdf

Life sciences study design

All studies must disclose on these points even when the disclosure is negative.

Sample size	Sample sizes were not predetermined by any statistical methods. For both cryo-EM datasets, around 3000 movies were collected, and the resulting particles for SaCas9-sgRNA-AcrIIA15 and SaCas9-sgRNA-AcrIIA15-IR are 3.7 M and 5.9 M, which are sufficient to generate high-resolution maps. For the crystal datasets, completeness over 99% shows that enough images have been collected. All the biochemical assays were conducted with at least two individually prepared protein samples.
Data exclusions	For cryo-EM analysis, poor quality cryo-EM images were removed. After 2D/3D classifications, particles in bad classes were discarded and the data processing flowchart were illustrated in the Supplementary figures. No data was excluded for biochemical assays.
Replication	The biochemical assays were repeated at least three times on different dates, giving similar results. The investigators confirm the reproducibility of these experiments.
Randomization	For the cyrstal and cyro-EM data collection and processing, samples were allocated randomly. For the biochemical assays, the data obtained from these experiments are objective and does not involve any subjective assessment by the experimenter, so randomization is not relvant to our study.
Blinding	Blinding is not relevant in this study, because all samples were subjected to extraction and purification steps, and they were definite during experiments.

Reporting for specific materials, systems and methods

We require information from authors about some types of materials, experimental systems and methods used in many studies. Here, indicate whether each material, system or method listed is relevant to your study. If you are not sure if a list item applies to your research, read the appropriate section before selecting a response.

Materials & experimental systems

n/a	Involved in the study
☐	☒ Antibodies
☒	☐ Eukaryotic cell lines
☒	☐ Palaeontology and archaeology
☒	☐ Animals and other organisms
☒	☐ Clinical data
☒	☐ Dual use research of concern
☒	☐ Plants

Methods

n/a	Involved in the study
☒	☐ ChIP-seq
☒	☐ Flow cytometry
☒	☐ MRI-based neuroimaging

Antibodies

Antibodies used: Commercial antibody: Anti-His antibody (1/20 dilution, cytiva, cat#29234602)

Validation: For anti-His antibody, validation was performed on surface plasmon resonance (SPR). Specific description can be found within the relevant method section.

Plants

Seed stocks: *Report on the source of all seed stocks or other plant material used. If applicable, state the seed stock centre and catalogue number. If plant specimens were collected from the field, describe the collection location, date and sampling procedures.*

Novel plant genotypes: *Describe the methods by which all novel plant genotypes were produced. This includes those generated by transgenic approaches, gene editing, chemical/radiation-based mutagenesis and hybridization. For transgenic lines, describe the transformation method, the number of independent lines analyzed and the generation upon which experiments were performed. For gene-edited lines, describe the editor used, the endogenous sequence targeted for editing, the targeting guide RNA sequence (if applicable) and how the editor was applied.*

Authentication: *Describe any authentication procedures for each seed stock used or novel genotype generated. Describe any experiments used to assess the effect of a mutation and, where applicable, how potential secondary effects (e.g. second site T-DNA insertions, mosiacism, off-target gene editing) were examined.*
